# In response to the editorial: "Brazil is getting older: some lessons from the Bambuí Health and Aging Study"

**DOI:** 10.1590/S1516-31802005000100010

**Published:** 2005-01-02

**Authors:** Luiz Eugênio Garcez Leme

The editorial on the aging pattern of Brazil’s population,^1^ indicating that the aging of our population is a fast, irreversible and progressive phenomenon, was very opportune. The real impact of this aging, especially the proportional increase in the number of very old people (more than 80 years old) will be felt over the next few decades. This will be due both to the changes in dependence and the consequential problems of this for social security, and to the changes in the profile of healthcare events. There will be concomitant multiplication of high-complexity events and accidental events, such as hip fractures, with high material and human costs.

It is indispensable that government, society and universities should quickly begin to make preparations for facing up to this challenge. Programs like the vaccination campaigns against influenza and the Statute of the Elderly are evident achievements. However, what is more important than declaring rights is to ensure that they are fulfilled.

We hope that this lucid editorial in São Paulo Medical Journal can serve as a further, very necessary cry of alert about the problematic situation of elderly people in Brazil.

## References

[1] Lotufo PA (2004). Brazil is getting older: some lessons from the Bambuí Health and Aging Study. São Paulo Med.

